# Enhanced Killing and Antibiofilm Activity of Encapsulated Cinnamaldehyde against *Candida albicans*

**DOI:** 10.3389/fmicb.2017.01641

**Published:** 2017-08-29

**Authors:** Shahper N. Khan, Shakir Khan, Jawed Iqbal, Rosina Khan, Asad U. Khan

**Affiliations:** Interdisciplinary Biotechnology Unit, Aligarh Muslim University Aligarh, India

**Keywords:** CNMA, multilamellar liposome, apoptosis, biofilm, *Candida albicans*

## Abstract

*Candida* sp. impelled opportunistic infection in immune-compromised patients ensuing from asymptomatic colonization to pathogenic forms. Moreover, slow spread of *Candida* species inducing refractory mucosal and invasive infections brings acute resistance to antifungal drugs. Hence, here we probed the effect of encapsulated preparation of cinnamaldehyde (CNMA) in multilamellar liposomes (ML) against *Candida albicans*. The efficacy of ML-CNMA against *Candida* biofilm was assessed by scanning electron microscopy, transmission electron microscopy, as well as light microscopy and its percent inhibition, was determined by XTT [2,3-bis-(2-methoxy-4-nitro-5-sulfophenyl)-2H-tetrazolium-5-carboxanilide] and crystal violet assay. ML-CNMA showed more fungicidal activity than free CNMA as well as multilamellar liposomal amphotericin B (ML-Amp B), which was further confirmed by spot test assay and Log-logistic dose–response analysis. Antifungal activity was driven by reactive oxygen species and cellular damage by sustained release of CNMA. Effect on hyphal formation during 48 h in presence/absence of ML-CNMA was observed under a microscope and further substantiated by RT-PCR by amplifying *HWP1*, the gene responsible for hyphal wall protein formation. Apoptotic programmed cell death was analyzed by FACS analysis which was further confirmed by cytochrome C release assay. This study elucidates the mechanistic insight of the enhanced antifungal activity of ML preparation of CNMA against *Candida* infections.

## Introduction

The threat of developing HIV-related complications differs with the extent of immunosuppression in patients (Wadhwa et al., 2007). In spite of the promising effect of antiretroviral therapy on the infectious complications of HIV/AIDS (Michelet et al., 1998), candidiasis stands alone as a major opportunistic infection in HIV-infected cases (Revankar et al., 1998; Morgan, 2005). *Candida albicans* is a pathogen, causing a multitude of oral and vaginal infections to severe systemic disorders in immune compromised patients (Fidel, 2006; Perlroth et al., 2007). It forms biofilms, an organized and highly structured community of cells (Jin et al., 2005; Ramage et al., 2005), which lately enhanced resistance against antimicrobial agents as well as to host defenses, with profound clinical implications (Costerton et al., 1999; Donlan, 2001; Khan and Khan, 2016). *Candida* infections are getting resistant to fluconazole and amphotericin B (Amp B; Pfaller, 1996; Mah and O’Toole, 2001).

It has been reported that on treatment of systemic and topical fungal infections with liposomal (Berenguer et al., 1994) or lipid (Caillot et al., 1993) formulations of antifungal drugs reduces Amp B toxicity and an increase in therapeutic index (Ralph et al., 1991). The earlier studies have revealed the existence of apoptotic cell death responses in yeast (Madeo et al., 1997; Shirtliff et al., 2009; Khan et al., 2012). In fact, various antifungal agents, such as Amp B (Phillips et al., 2003), have already been illustrated to act as yeast-specific apoptotic programmed cell death-inducer. Mitochondria playing the central role in apoptosis, and the inhibition of the mitochondrial electron transport chain, leads to consequent discharge of reactive oxygen species (ROS), as an early event in apoptotic cell death (Westwater et al., 2005; Zhang et al., 2005).

Cinnamaldehyde (CNMA) is a major constituent of *Cinnamomum zeylanicum* (Lauraceae) extract, which is a widely used flavoring compound that has traditionally been used to treat human diseases, including dyspepsia, gastritis, and inflammatory diseases. Studies have demonstrated that CNMA exhibits antioxidant (Molania et al., 2012), anti-inflammatory (Kang et al., 2016), and anticancer activity (Lin et al., 2013), and the compound has been classified as a generally recognized as safe (GRAS) molecule for food preservation by the United States Food and Drug Administration (Wei et al., 2011). CNMA has proven to be effective against some species of toxicogenic fungi and also against respiratory tract pathogens, including *Aspergillus, Candida*, and *Cryptococcus* (Shreaz et al., 2016). The restrictive factors for these compounds are their high volatility, sparingly water solubility, and the irritant effect that make difficult to formulate preparations containing them. Therefore, encapsulating CNMA in lipid multilayer could address some of these concerns by preventing their degradation, control release rate, improve bioavailability, lowering dose frequency, and enhanced antimicrobial activities. Hence, we initiated our study to demonstrate the antibiofilm and apoptotic effect of encapsulated multilamellar liposomes (ML)-CNMA on *C. albicans*.

## Materials and Methods

### Growth Condition of Microorganism

*Candida albicans* ATCC 24433 and clinical isolates (i.e., CA11, CA5, CA3, and CA16) were grown in yeast peptone dextrose (YPD) medium; 1% yeast extract, 2% peptone, 2% dextrose (HiMedia) at 37°C overnight. Cells were harvested and washed twice with sterile phosphate-buffered saline (PBS; 10 mM phosphate buffer, 2.7 mM potassium chloride, 137 mM sodium chloride; pH 7.4; Jin et al., 2003). Cells were re-suspended in RPMI 1640 supplemented with L-glutamine and buffered with morpholine propane sulfonic acid (MOPS) (HiMedia) and adjusted to the desired density for antibiofilm assay [10^7^ cells⋅ml^-1^ at 0.1 optical density (OD), absorption at 600 nm] and also counted with a hemocytometer (Chandra et al., 2001). The cell density maintained at 0.5–2.5 × 10^3^ cells⋅ml^-1^ (in RPMI 1640 medium with L-glutamine with MOPS) for the broth microdilution susceptibility assay conferring to CLSI M27-A2 procedure (Park et al., 2006).

### Liposomes Preparation

The “hand shaking” method was used for the preparation of ML-CNMA and ML-Amp B (Trafny et al., 1995). At 37°C temperature, 100 mg sample of phosphatidylcholine (Sigma–Aldrich, India) and 50 mg of cholesterol (Sigma–Aldrich, India) were suspended in 5 ml of chloroform, in the round bottom flask connected with rotary evaporator. The evaporation process continued until dry film visualization. The residual solvent was removed by vacuum evaporation for 1 h, and then 5 ml of Amp B (200–900 mg⋅l^-1^; dissolved in 1:1 of 1% DMSO:PBS) and CNMA (0.50–1 mg⋅l^-1^; dissolved in 1% DMSO) were added for the synthesis of ML-Amp B and ML-CNMA, respectively. The rotatory flask was evaporated at 60 rpm for 30 min at room temperature and then put in the stagnant position for 2 h. The liposomal non-entrapped Amp B and CNMA was separated by overnight dialysis by corresponding 1% DMSO:PBS and 1% DMSO solvents, respectively. For the quantification of entrapped drugs, liposomes were ruptured by treatment with 0.1% sodium deoxycholate and released drugs concentration were calculated by comparison with standard calibration graphs (for released Amp B; calculations based on UV-Vis absorption vs. drug concentrations, see Supplementary Figure S1, and for released CNMA; calculations based on colony forming unit [CFU = log_10_ (cells⋅ml^-1^) vs. CNMA concentrations], see Supplementary Figure S2). ML-CNMA and ML-Amp B was used throughout the study. The central values of synthesized liposomes in mean ± SD size were measured by Malvern Zetasizer Nano ZS (Malvern, Southborough, MA, United States).

### Determination of Minimum Inhibitory Concentration

*Candida albicans* were found sensitive to the Amp B/ML-Amp B and CNMA/ML-CNMA fraction. For minimum inhibitory concentration (MIC), serial dilutions of the CNMA (initial concentration 1 g⋅ml^-1^) and Amp B (initial concentration 1 mg⋅ml^-1^) were performed according to CLSI M27-A2 procedure (Park et al., 2006). The range of liposomes that have different drugs’ concentrations (i.e., quantified by calibrated UV-Vis and CFU. for ML-Amp B and ML-CNMA, respectively) used to identify the liposomal drugs MIC values. Each fungal inoculum was prepared in normal saline and density was adjusted to 0.1 OD; 0.5–2.5 × 10^3^ cells⋅ml^-1^ (absorption at 600 nm). Microtiter plates were incubated incubator (Thermo Fisher Scientific incubator) at 37°C and the MIC was recorded after 24–48 h. Proper controls of dissolvent (i.e., solvent of drugs) as blank control, fungal inoculum as positive control and empty lipid carrier as negative control were used throughout the study.

### Spot Test for Drug Susceptibility and Drug–Cell Viability Analysis

*Candida albicans* ATCC 24433 was further rechecked for their resistance to free and ML-Amp B as well as ML-CNMA by spot test. Strains were grown overnight on YPD plates at 37°C. Cells were then suspended in normal saline to 0.1 OD (absorption at 600 nm). A 5 μl of fivefold serial dilutions of each yeast culture was spotted onto YPD plates in the absence (control) of any drug and presence of free and ML-Amp B as well as ML-CNMA. Growth differences were recorded after incubation of plates for 48 h at 37°C. Growth was not affected by the presence of the controls of dissolvent (i.e., solvent of drugs) as blank control and empty lipid carrier as negative control used for the drugs. The dose–response (drug–cell viability) was used to determine the effective concentrations of the drug (*E*) for percentage decrease in *Candida* cell viability (CV). This *in vitro* method used to predict the exact concentration of drug that may effective for the *in vivo* model (Hawser and Douglas, 1995; Larsen et al., 2005). The data; % percentage values of viability corresponding to used drug concentrations were determined by CFU. Nonlinear regression was applied to the data from the dose–response experiments using the statistical freeware program R 3.2.4 with the *drc* package (Ritz et al., 2015). A four-parameter equation (Eq. 1) was used to describe the dose–response curves. The effect of ML-CNMA on the CV was assumed to follow a log-logistic four parameter model (Streibig et al., 1993).

(1) $$\mathrm{CV}(x)=C+\frac{D-C}{1+\exp(b(\log(x)-\log(E_{\%})))}$$

The CV denotes the viability of *C. albicans* cells as a function of the concentrations of drugs (*x*). The *D* and *C* are the upper and lower asymptotes of CV at 0 and maximum drugs, respectively. The calculations drugs concentrations (denoted by *E*) are corresponding of the reduced CV (%) (midway between the *D* and *C*). The slope of the curve at *E*_%_ is proportional to *b*.

### *Candida* Biofilm Formation

Biofilms were produced on commercially available pre-sterilized, polystyrene, flat-bottom 96-well microtiter plates (TPC96, HiMedia, India) as reported earlier (Kuhn et al., 2002). As controls, three wells of each microtiter plate were handled in an identical fashion, except that no *Candida* suspension was added. Following the adhesion phase (i.e., 10^7^ cells incubated in 100 μl RPMI media for 90 min at 70–150 rpm at 37°C), the cell suspensions were aspirated and each well was washed twice with 150 μl of PBS to remove loosely adherent cells. A total of 100 μl of RPMI 1640 was then transferred into each of the washed wells with a pipette, and the plates were incubated at 37°C in a shaker at 75 rpm. The biofilms were allowed to develop for 24 and 48 h and then the yeasts were visualized under a microscope and quantified by XTT [2,3-bis-(2-methoxy-4-nitro-5-sulfophenyl)-2H-tetrazolium-5-carboxanilide] and crystal violet assays. All assays were carried out on three different occasions in triplicate with proper controls of dissolvent (i.e., solvent of drugs) as blank control, fungal inoculum as positive control and empty lipid carrier as negative control were used throughout the study (Jin et al., 2003).

### Crystal Violet Staining

*Candida albicans* biofilm was assessed by microdilution method and quantified by a slight modification of a crystal violet assay as described earlier (Jin et al., 2003). A six different concentrations (550, 500, 450, 400, 350, 300 mg⋅l^-1^) of CNMA as well as six different concentrations (300, 240, 155, 80, 56, 30 mg⋅l^-1^) of CNMA (@/in ML) were used to treat the *Candida* biofilm. And also, a five different concentrations of Amp B (0.45, 0.40, 0.35, 0.30, and 0.25 mg⋅l^-1^) as well as five different concentrations of Amp B (0.60, 0.55, 0.50, 0.45, and 0.40 mg⋅l^-1^) @/in were used to treat the *Candida* biofilm. The biofilm developed in microtiter plates were twice washed with 200 μl PBS and then air dried for 45 min. The wells were stained with 110 μl of 0.4% aqueous crystal violet (Sigma–Aldrich, India) solution for 45 min. Afterward, washing was done four times with 350 μl of sterile distilled water and subsequently destained with 200 μl of 95% ethanol. After 45 min of destaining, 100 μl of the destaining solution was transferred to a new well and the amount of the crystal violet stain in the destaining solution was measured with a microtiter plate reader (Bio-Rad Laboratories) at 595 nm. The absorbance values for the controls were subtracted from the values for the test wells to minimize background interference. The percentage of biofilm reduction was calculated by the following equation.

(2) $$\mathrm{Biofilm}\;\mathrm{reduction}(\%)=\left(1-\frac{{\mathrm{OD}}_{\mathrm{treated}}@595\mathrm{nm}}{{\mathrm{OD}}_{\mathrm{control}}@595\mathrm{nm}}\right)\times 100\%$$

### XTT Assay

This assay was performed by previously described methods (Jin et al., 2003; Kuhn et al., 2003). A 1 mg⋅l^-1^ XTT (Sigma, MO, United States) solution was prepared in PBS. The XTT was filtered and sterilized using a 0.22 μm-pore-size filter and then stored at -70°C until required. Menadione (Sigma, MO, United States) solution (0.4 mM) was also prepared and filtered immediately before each assay. Prior to each assay, XTT solution was thawed and mixed with menadione solution at a volume ratio of 20:1. The adherent cells were washed four times with 200 μl of PBS to remove loosely adherent cells. Afterward, 158 μl of PBS, 40 μl of XTT, and 2 μl of menadione were inoculated to each of the prewashed wells. After incubation in the dark for 2 h at 37°C, 100 μl of the solution was transferred to a new well and a colorimetric change in the solution was measured using a microtiter plate reader (Bio-Rad Laboratories) at 490 nm.

### Microscopic Study

Treated and control Biofilms were scraped, re-suspended in sterilized distilled water containing one drop of 50 mg⋅l^-1^ propidium iodide (PI; Sigma) as well as one drop of 0.0025% Fluorescent Brightener 28 (Sigma Chemical, St. Louis, MO, United States) incubated for 10 min at room temperature in dark (Harrington and Williams, 2007). The fluorescents probed biofilm/cells was washed with distilled water three times. The samples were examined under the light microscope equipped with differential interference contrast (Nomarski) and fluorescence capabilities (IX-81, Olympus). For SEM analysis, *C. albicans* cells were grown on poly-L-lysine (Sigma) coated glass coverslip discs (HiMedia, India) in 12-well cell culture plates (CoStar, Bethesda, MD, United States). The glass cover slips coating with poly-L-lysine (2% wt/vol) were done according to described method by Dong et al. (2011). This adherent coatings were sterilized for 1 h. in laminar air flow equipped with UV radiation. According to SEM sample protocol (Chandra et al., 2008), here, standardized cell culture (2 ml of a suspension containing 10^7^ cells⋅ml^-1^ in RPMI-1640) were used in each culture plate well and adhered to glass cover slip for 2 h. Cells were treated with ML-CNMA (80, 240, and 300 mg⋅l^-1^) and incubated at 37°C. Each disc was removed from culture plate and wash with sterilized PBS. Samples were then dried in a desiccator after treatment of serial ethanol dehydration (at 25, 50, 65, 95, and 100% ethanol; for 15 min difference in each steps). The samples were sputter coated with gold by cathodic spraying and observed by scanning electron microscope (LEO 435 VP) at 15KX and 20 KX magnification (Bandara et al., 2010). For TEM, culture material processed according to Bozzola and Russell (1999). Ultra-thin sections of the cells were stained with uranyl acetate and lead citrate and observed under Morgagni 268 (D) transmission electron microscope at 50 KX magnification.

### Confocal Laser Scanning Microscopy

The effects of ML-CNMA on *Candida* cells during the biofilm formation were analyzed by confocal laser scanning microscopy (CLSM; Chandra et al., 2008). *Candida* cells were treated with ML-CNMA and incubated at 37°C for 12 h on glass coverslips. After that, these cover slips were removed and transferred to new six-well culture plates and incubated for 45 min at 37°C in 4 ml of PBS containing the following fluorescent dyes as molecular fluorescent probes. These fluorescent probes are concanavalin A (Con A) Alexa Fluor 488 conjugated, 25 μg⋅ml^-1^; Invitrogen, Carlsbad, CA, United States), FUN-1 (Fungolight 10 μM; Invitrogen), and 4,6-diamidino-2-phenylindole (DAPI, 2.5 μg⋅ml^-1^, Sigma–Aldrich). After incubation with probes, coverslips flipped on glass plates and observed with a FluoView FV1000 (Olympus, Tokyo, Japan) confocal laser scanning microscope equipped with argon and HeNe lasers.

### Rate of ROS Generation

ML-CNMA/CNMA induced ROS generation rate was quantified by an indirect method, where produced ROS oxidized the cytoplasmic dichlorofluorescein diacetate (DCFH-DA) to fluorescent dichlorofluorescein (DCF). Initially, *Candida* cells treated with ML-CNMA/CNMA for 60 min. Subsequently, a 10 mM DCFH-DA was added and incubated for 10, 15, 30, and 60 min in 2 ml well microtiter plate at 37°C. The produced cytoplasmic DCF fluorescence was measured (at »_em_ = 522 nm) following excitation with »_ex_ = 490 nm light. The quantity of ROS production was proportional to the amount of fluorescence intensity produced by DCF (emission = 520 nm) from the cells. Fluorescent intensity of fluorescent probe was measured by Hitachi (Tokyo, Japan) F-4500X fluorescence spectrometer.

The cytoplasmic DCF fluorescence formation can be expressed as Eq. 3 (Daghastanli et al., 2008). Here, *F*_(_*_t_*_)_ is the intensity of fluorescence at time *t, F*_0_ is the fluorescence at time 0, *b* is a fit parameter, and *k* is the time constant.

(3) $$F_{\left(t\right)}=F_{0}\left(1-\;\beta e^{-t/k}\right)$$

Hence, the rate of DCF formation r_(_*_t_*_)_ can be measured from d(*F_t_*)/dt (Eq. 4).

(4) $$r(t)=\frac{d(F_{t})}{\mathrm{dt}}=\frac{d}{\mathrm{dt}}\left[F_{0}-F_{0}\;\beta e^{-t/k}\right]\;\left(4\right)$$

### Apoptosis by FACS Analysis

PI (Sigma–Aldrich) and annexin V-FITC (Sigma–Aldrich) probes were used to assess the cellular integrity and externalization of phosphatidylserine (PS), respectively. Here, PS is an early marker of apoptosis and probed by annexin V-FITC when it exposed on the plasma membrane’s outer surface. Initially, *C. albicans* cells were protoplasted, where cells (10^7^ cells⋅ml^-1^) was washed twice in PBS and incubated at 30°C for 30 min in presence of 0.5 ml (pH 7.2) of 50 mM K_2_HPO_4_ + 5 mM EDTA + 50 mM DTT. The incubated cells digested by 0.5 ml (pH 7.2) of 50 mM KH_2_PO_4_ + 40 mM 2-mercaptoethanol + 3 μg⋅ml^-1^ chitinase (Sigma–Aldrich) + 1.8 μg⋅ml^-1^ lyticase + 12 μl glucuronidase + 0.15 mg⋅ml^-1^ zymolyase + 20 μl of glusulase in 2.4 M sorbitol. This digestion process takes place for 45 min at 30°C. Protoplasts (10^7^ cells⋅ml^-1^) were washed in modified annexin binding buffer (10 mM HEPES/NaOH, pH 7.4; 40 mM NaCl + 50 mM CaCl_2_ + 1.2 M sorbitol). The protoplasted cells were used for treatment with ML-CNMA (80, 155, and 240 μg⋅ml^-1^) in RPMI 1640 media supplemented with MOPS (pH 7.4) in the culture flask. After 4 h incubation at 30°C with shaking (75 rpm.), cells were harvested and assessed for apoptosis. Annexin-V binding assays were performed according to the protocol of Madeo et al. (1999), in modified annexin binding buffer containing 20 μl⋅ml^-1^ annexin reagent and 5 mg⋅l^-1^ PI. The annexin and PI status of protoplasts were recorded for each treatment, and each assay was repeated in triplicate.

### Cytochrome C Assay

The extent of apoptosis was indirectly quantified by relative concentrations of cytochrome C (Cyt C) produced in the treated cells’ cytoplasm/mitochondria. Here, mitochondria were isolated by the method of Wu et al. (2010). *Candida* cells were grown in YPD broth at 37°C to early stationary phase and quantified by dilution up to 1 × 10^7^ cells⋅ml^-1^ with normal saline and further incubated with ML-CNMA (240 mg⋅l^-1^) at 30°C for 24 h. The cells were washed in PBS (7.2 pH) as well as centrifuged at 5000 × *g* for 5 min. The pellet decanted in the homogenization medium [50 mM Tris (pH 7.5), 2 mM EDTA, and 1 mM phenylmethylsulfonyl fluoride], and supplemented with 2% glucose. Initially, cell debris and unbroken cells were removed from the homogenization medium by 2000 × *g* centrifugation for 10 min and, further supernatants were collected after centrifugation at 30,000 × *g* for 45 min. This supernatants were used for Cyt C quantification (i.e., released from mitochondria to cytoplasm). The pellet was resuspended in 50 mM Tris (pH 5.0) + 2 mM EDTA and incubated for 5 min at 37°C, and centrifuged at 5000 × *g* for 30 s. This pellet was used for the determination of Cyt C remaining in mitochondria. Protein estimations (of supernatant as well as pallets) were done according to the method of Bradford (1976), using BSA as the standard. The supernatant and pallets being reduced by 500 mg⋅l^-1^ ascorbic acid at room temperature for 5 min and the amount of Cyt C in supernatants and mitochondria were determined by measuring absorbance at 550 nm with a Spectrofluorophotometer (Shimadzu UV-1700).

### RNA Extraction from CNMA/ML-CNMA Treated *Candida* Cells and RT-PCR

The *Candida* cells (ATCC 24433) were treated with CNMA as well as ML-CNMA. The cells (that include positive control/treated and negative control/non-treated) were pelleted and RNA was extracted by using RNA extraction kit (YeaStar RNA Kit, Zymo Research). Pure RNA was quantified and used to synthesize first strand cDNA as manufacturer’s instruction (cDNA synthesis kit, Fermentas, United States). PCR reactions were performed by using the primers *HWP1* F 5′-CCA CTA CTA CTG AAG CCA AAT C-3′; *HWP1* R 5′-AAG TGG ATA CTG TAC CAG TTG G-3′; *EFB1* F 5′-AGT CAT TGA ACG AAT TCT TGG CTG-3′ and *EFB1* R 5′-TTC TTC AAC AGC AGC TTG TAA GTC-3′. The following conditions were used for amplification of *HWP1*: 94°C for 5 min, 94°C for 30 s, 56°C for 30 s, 72°C for 30 s, and 72°C for 30 s for 30 cycles and for housekeeping gene; primer *EFB1*: 94°C for 30 s, 60°C for 30 s, and 72°C for 60 s (21 cycles), respectively (Davis-Hanna et al., 2008).

### Modeling and Docking Simulation

The amino acid sequence of 14-α demethylase for *C. albicans* was extracted from Swissprot database. As the crystal structure of 14-α demethylase of *C. albicans* was not available within Protein databank, so its 3D model was generated. Blastp search against PDB was performed to search for a suitable template. Crystal structure of human lanosterol 14α-demethylase (CYP51) (PDB id: 3LD6) having 40% identity with the target was taken as a template for constructing the 3D protein model. Modeler 9v8 was used for building the 3D structure of 14-α demethylase. The coordinates of HEM group were transferred from the template to the generated structure. The model generated was further refined to remove the bad steric clashes by using CharmM force field. Furthermore, the refined structure was validated through Procheck. AmpB and CNMA were docked into the active site of 14-α demethylase using LigandFit docking program (Venkatachalam et al., 2003; Khan et al., 2008).

### Statistical Analysis

The significance and central values of effects (after the treatment of ML-CNMA/Amp B) were analyzed by open source statistical software R (Comprehensive R Archive Network; CRAN) (R Development Core Team, 2013). Differences between mean values were assessed either by Student’s *t*-test or by one-way analysis of variance (ANOVA), followed by a Newman–Keuls *post hoc* test. Values of *P* < 0.05 and *P* < 0.001 were considered to be significant. Bar graphs and other plots were prepared by ggplot2 in R. Graph analysis were done by packages (“ggplot2”) downloaded in R console.

## Results

### Susceptibility Test

**FIGURE 1:**
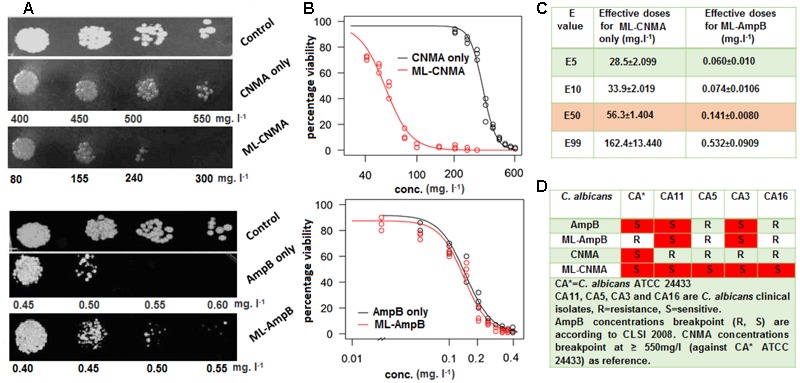
**(A)** Drug resistance profiles of *Candida albicans* ATCC 24433 determined by the spot assay. Growth was not affected by the presence of the solvents used for the drugs. **(B)** Log-logistic dose–response curve illustrating the growth inhibitory effect of both cinnamaldehyde and AmpB in presence and absence of ML. **(C)** The corresponding table shows the effective dose or concentration of drug (ML-entrapped) for particular percentage inhibition (*E*_%_) in cell viability (data derived by log-logistic dose–response curve analysis). Dissolvent and empty lipid carrier was used as controls. **(D)** Table shows Susceptibility and resistant profile of drugs/ML-entrapped drugs against various clinical isolates.

The effective concentrations of liposome entrapped Amp B and CNMA were calculated by the calibration graphs, where released drugs concentrations compared with UV-Vis absorptions and CFU values, respectively (see Supplementary Figures S3, S4). The MIC values of Amp B and CNMA alone against *C. albicans* ATCC 24433 are 0.50 and 550 mg⋅l^-1^, respectively. Whereas, MICs of ML-Amp B and ML-CNMA are 0.55 and 240 mg⋅l^-1^, respectively. Hence, *Candida* strain ATCC 24433 is merely equal sensitive to the free Amp-B compared to the ML-Amp B whereas, ML-CNMA departed more sensitivity than free CNMA. Further the spot test assay also corroborates enhance fungicidal effects of ML-CNMA as compared to CNMA alone (**Figure 1A**). To further understand the role of the liposome in CV of *C. albicans*, we fitted a dose–response curve to ascertain the cellular viability (**Figures 1B,C**). Interestingly, our findings showed the necessary concentrations of CNMA (in ML-CNMA formulation) to reduce the 5, 10, 50, and 99% CV is lower (∼28.6, 34, 56, and 162 mg⋅l^-1^) as compared CNMA alone (∼235, 257, 333, and 572 mg⋅l^-1^). The same trends also departed by ML-CNMA against clinical isolates (see Supplementary Table S1). Contrary, the same liposome formulation for Amp B showed the opposite result, where the necessary concentrations of Amp B to reduce the 5, 10, 50, and 99% CV is about (∼0.060, 0.074, 0.141, and 0.532 mg⋅l^-1^) almost same as compared to Amp B alone (∼0.058, 0.074, 0.147, and 0.621 mg⋅l^-1^). The comprehensive susceptibilities/resistant of clinical isolates were enlisted in the tabular form (**Figure 1D**), where MIC break point of Amp B taken according to CLSI 2008 guideline (Clinical and Laboratory Standards Institute, 2008) and CNMA break point was ≥550 mg⋅l^-1^ (MIC against *C. albicans* ATCC 24433).

### Mode of Action of ML-CNMA

**FIGURE 2:**
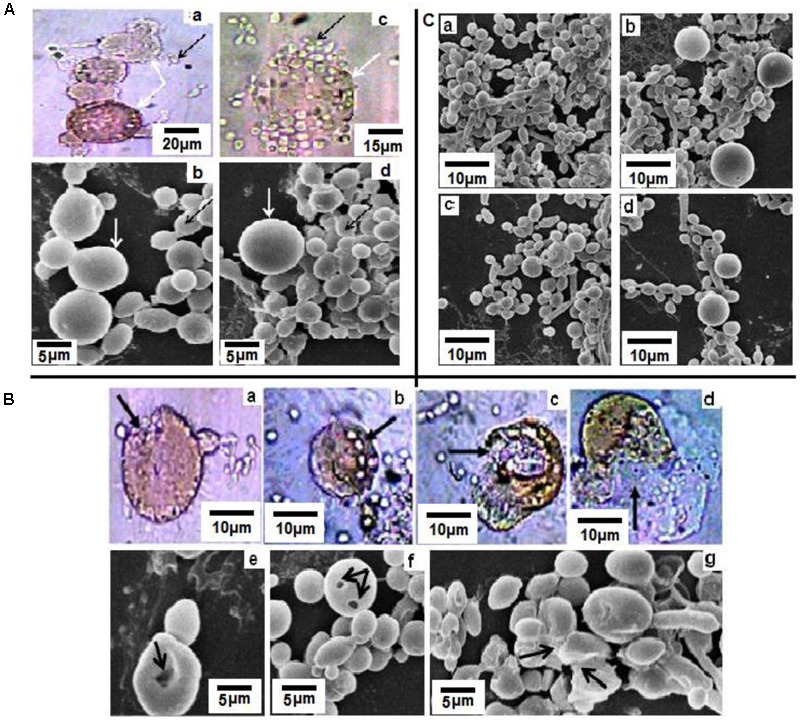
**(A)** Light **(a,c)** and scanning electron microscopic **(b,d)** studies of *Candida albicans* and ML-CNMA (300 mg⋅l^-1^ CNMA entrapped ML-CNMA) interaction. Panels **(a,b)** showed interaction among liposomes, whereas panels **(c,d)** showed the interaction between the liposome and *Candida* cells. White arrows indicate liposome whereas black arrows indicate *Candida* cells. **(B)** Panels **(a,e)** showed channels/grooves formed in liposome after attachment of cells, and later elongated *Candida* cells attached liposome **(b,f)** formed more grooves. Finally, liposome ruptured **(c,d,g)** causing release of CNMA, indicated by different black arrows. **(C)** Control biofilm **(a)** were treated with ML-CNMA **(b–d)** with increasing concentration (30, 80, and 300 mg⋅l^-1^) for 24 h at 37°C. Dissolvent and empty lipid carrier was used as controls.

The adhered cells were treated with ML-CNMA (i.e., 300 mg⋅l^-1^ CNMA entrapped ML-CNMA) for 24 h and were investigated under light and scanning electron microscope (**Figures 2A,B**). Prior to that, the size distribution of the synthesized ML-CNMA was 2.7 ± 3 μm size (measured by Malvern Zetasizer Nano ZS; see Supplementary Figure S3). In addition, the light microscope also showed the synthesized ML-CNMA with average size ∼3 μm (see Supplementary Figure S4). Here, the micrographs show the interaction among the liposomes (**Figures 2Aa,b**) as well as between liposome and *Candida* cells (**Figures 2Ac,d**). Interestingly, after the treatment of biofilm, the size of liposomes increases from ∼6.8 to ∼15.2 μm during the gap of 2–8 h time period (Supplementary Figure S5). Moreover, SEM images showed a very unambiguous affinity of binding between liposome–liposome and liposome–*Candida* cells. A sort of groove was observed on contact of cells with liposome (**Figures 2Ba,e**) through which cells entered into the liposome (**Figures 2Bb,f**) and came in further contact with drug and eventually liposome got ruptured after interaction with large number of *Candida* cells (**Figures 2Bd,g**). Further, its antifungal effect on *Candida* biofilm was observed by treatment with varying concentrations of ML-CNMA (0, 30, 80, and 300 mg l^-1^) for 24 h. SEM analysis showed the effects of ML-CNMA (**Figures 2Cb–d**) on *C. albicans* biofilm formation. The images (**Figures 2Cc,d**) showed remarkable biofilm reduction in the presence of ML-CNMA.

### Biofilm Reduction

**FIGURE 3:**
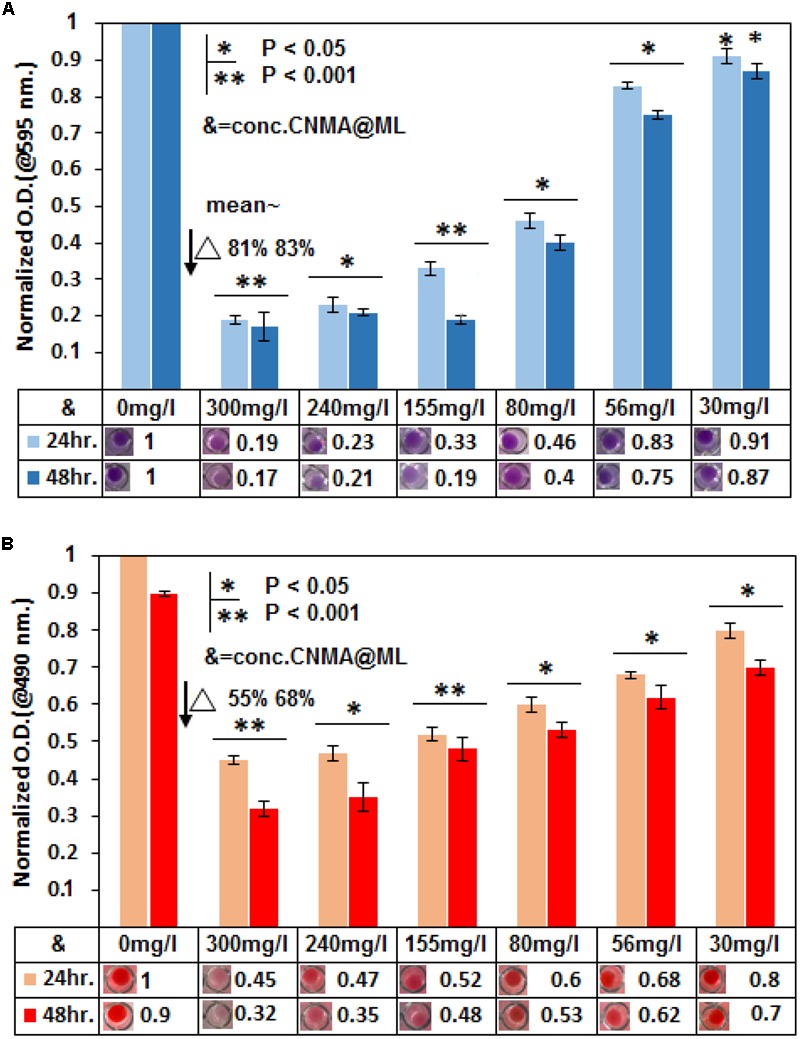
Reduction in biofilm formation was assessed by crystal violet assay and XTT. In crystal violet assay **(A)** as well as in XTT **(B)**, six different concentrations (300, 240, 155, 80, 56, and 30 mg⋅l^-1^) of CNMA (@/in ML) were used to treat the *Candida* biofilm and results were normalized with an untreated sample. Control (untreated) and treated biofilm were grown for 24 and 48 h. The final values were mean optical density (OD) at 595 and 490 nm, respectively. Error bars indicate the standard error of the mean of three independent experiments performed in triplicates. Asterisks represent the significant difference in percentage biofilm reduction as compared with control, ^∗^*P* < 0.05, ^∗∗^*P* < 0.001.

Further, antibiofilm activity of ML-Amp B and ML-CNMA was observed using crystal violet and XTT reduction assay. Our study revealed that in crystal violet assay, maximum percent inhibition of biofilm formation observed during 24 and 48 h treatment was ∼81 and ∼83% by ML-CNMA (300 mg⋅l^-1^), respectively (**Figure 3A**). Whereas, ∼28 and ∼45% inhibition was found in the presence of ML-Amp B (0.45 mg⋅l^-1^) at 24 and 48 h treatment, respectively (Supplementary Figure S6). We have also employed tetrazolium salt (XTT) reduction assay for quantitative measurement of *Candida* biofilm formation, as it is more sensitive techniques to study the antifungal activity. Our data showed maximum percent inhibition as ∼55 and ∼68% with the highest time-dependent response at 300 mg⋅l^-1^ of ML-CNMA on 24 and 48 h treatment, respectively (**Figure 3B**). Whereas, ∼35 and ∼62% inhibition was observed Amp B (0.45 mg⋅l^-1^) treatment at 24 and 48 h treatment, respectively (Supplementary Figure S6). Furthermore, comprehensive crystal violet and XTT biofilm reduction assay findings of ML-CNMA against clinical isolates were enlisted in the tabular form (Supplementary Table S2). The ML-CNMA departed the same effect against the clinical isolates as reference *C. albicans* ATCC 24433 biofilm.

### Cyto-Metabolic Effects of ML-CNMA on *C. albicans*

**FIGURE 4:**
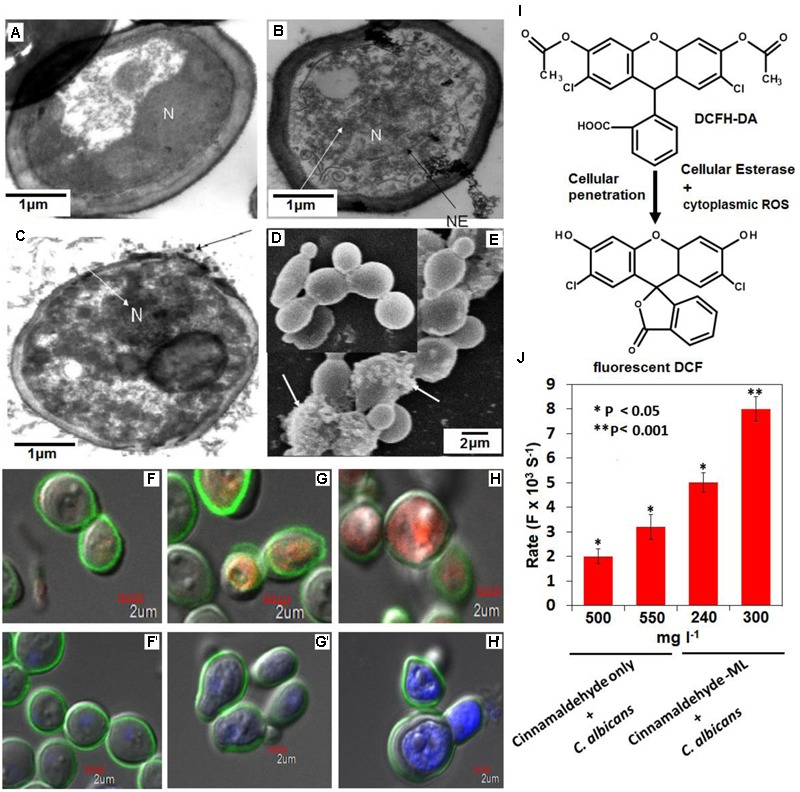
Probing cytometabolic outcomes of ML-CNMA treatment on *Candida* cells. Electron transmission micrographs of *Candida* cells grown for 12 h shows normal *Candida* cells (without any treatment) **(A)**, and intermediate stages **(B,C)** after treatment with ML-CNMA (240 and 300 mg⋅l^-1^), which eventually leads to apoptotic programmed cell death. Panel **(B)** shows chromatin condensation (white arrow) and nuclear envelope (NE) in the black arrow. Panel **(C)** shows the tiny vesicles on the outer face of the plasma membrane (black arrow) as well as chromatin condensation (white arrow). N is the nucleus. Panel **(E)** shows SEM of an apoptotic *Candida* cell treated for 36 h with ML-CNMA (300 mg⋅l^-1^), where surface blebbing is evident (white arrow) a sign of apoptosis in comparison to untreated cells **(D)**. The panels **(F–H)** stained with FUN1 and ConA, whereas **(F′–H′)** stained with DAPI and ConA. Though panels **(F,F′)** depict untreated cells but panels **(G,G′,H,H′)** represent treated cells (80 and 240 mg⋅l^-1^). **(I,J)** Quantification of (ROS-induced) cytoplasmic DCF green fluor generation. The enhancement of fluorescence rate (*F* × 10^-3^⋅S^-1^); corresponding to ROS production after the treatment of CNMA in presence and absence of ML. Error bars indicate the standard error of the mean of three independent experiments performed in triplicates. Asterisks represent the significant difference in florescence rate compared with control, ^∗^*P* < 0.05, ^∗∗^*P* < 0.001.

TEM has been used to reveal definitive ultra-structural features of the chromatin in apoptotic multicellular organisms. Control cells showed normal cellular morphology with a distinct cell wall and an intact nucleus (**Figure 4A**). In contrast, cells exposed to ML-CNMA (240 and 300 mg⋅l^-1^) manifested extensive chromatin condensation as aggregates in the nuclear envelope and tiny vesicles on the outer surface of the plasma membrane, which are the typical marker of apoptosis (**Figures 4B,C**). *Candida* cells when observed by SEM in the presence of ML-CNMA (300 mg⋅l^-1^) for 36 h, showed evident surface blebbing (white arrow), a sign of cell death (**Figure 4E**) as compared to normal cells (**Figure 4D**). Further, CLSM was performed to assess cellular metabolic activity, viability, and cell wall integrity using FUN1, Con A, and DAPI fluorescent probes, respectively. FUN1 gets transformed from yellow to the orange-red fluorescent intravacuolar structure by active or viable cells while Con A gives green fluorescence on binding with glucose and mannose moieties of the cell wall and DAPI provides blue fluorescence on intercalation with AT-rich regions of DNA. The untreated/control samples (**Figures 4F,F**′) show intact cell wall of green color due to Con A binding along with intra-vacuolar structure in focused orange-red fluorescence and blue color condensed nucleus for FUN1 and DAPI probes, respectively. Whereas, samples of *Candida* cells treated with 80 and 240 mg⋅l^-1^ ML-CNMA (**Figures 4G,H**), showed cytosolic diffused yellow and orange fluorescence, confirming vacuolar disintegration and fragmentation accompanied by the loss of cell wall integrity as depicted by fading green Con A fluor in metabolic cells. Likewise, DAPI stained treated samples also showed nuclear fragmentation as sprinkled and scattered blue fluor signals along with cell wall degeneration (**Figures 4G′,H′**). The non-fluorescent DCFH-DA (2′,7′-dichlorodihydrofluorescein) compound easily penetrates the cell membrane and converts to 2′,7′-DCF, a strong fluorescent molecule by ROS-mediated oxidation (Soh, 2006; Daghastanli et al., 2008; **Figure 4I**). The enhanced DCF fluorescence (*F*) is tantamount to ROS formation, hence the rate of fluorescence (*F* × 10^-3^⋅S^-1^) by ML-CNMA treatments are ∼2.5 times higher (for CNMA concentration 240 and 300 mg⋅l^-1^) as compared to CNMA alone (**Figure 4J**).

### Apoptotic Potential of ML-CNMA on *C. albicans*

**FIGURE 5:**
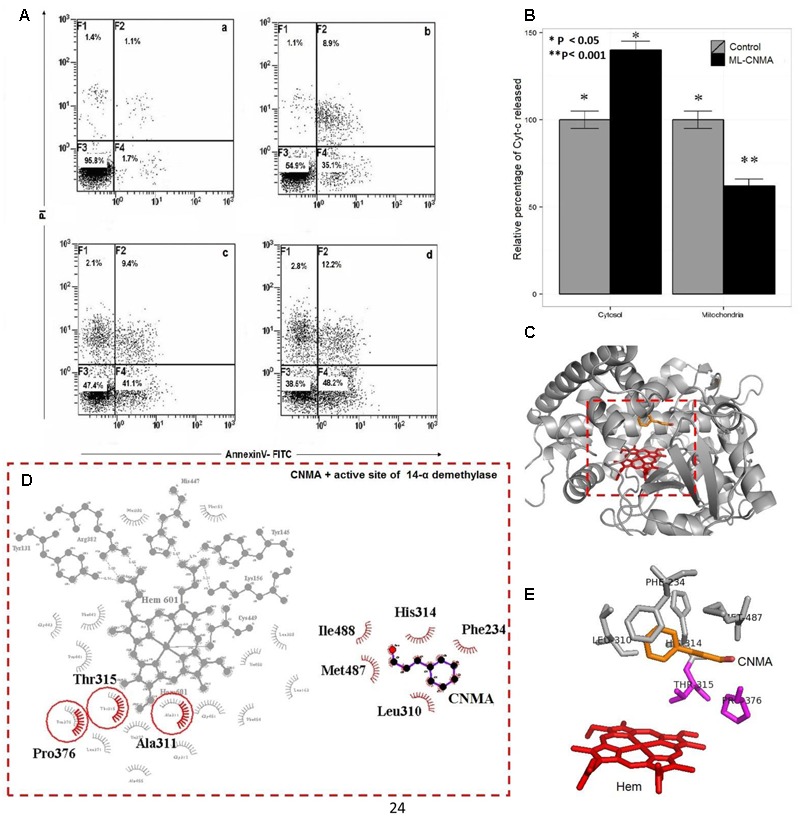
**(A)** *Candida albicans* was treated with ML-CNMA for 2 h: panel **(a)** is control (without any treatment) whereas panels **(b–d)** were treated with 80, 155, and 240 μg/ml of ML-CNMA. The figure shown was from a representative experiment repeated three times with almost similar results. **(B)** The effect of ML-CNMA on Cyt C release from mitochondria to cytosol in *C. albicans*. Concentrations of ascorbic acid-reduced Cyt C in mitochondria and cytosol were determined by measuring absorbance at 550 nm with a spectrofluorophotometer (Shimadzu). (■) Control, without any treatment and cells exposed to ML-CNMA (240 mg⋅l^-1^) were observed. **(C,D)** The Molecular modeling representation of 14-α demethylase active site and its binding interactions with CNMA. **(E)** The most common amino acids involved in the hydrophobic interactions (Thr315, Pro376, Met487, Leu310, His314, and Phe234) with Azole drug (such as Fluconazole) are also involved in with CNMA. Error bars indicate the standard error of the mean of three independent experiment performed in triplicate was considered significance, ^∗^*P* < 0.05, ^∗∗^*P* < 0.001.

Cells were labeled with annexin V and PI to analyze the apoptotic potential of the ML-CNMA formulation. The early and late apoptosis in presence of ML-CNMA (80, 155, and 240 μg⋅ml^-1^) were recorded as 35.1, 41.1, 48.2% and 8.9, 9.4, 12.2%, respectively (**Figure 5A**). Whereas in the presence of free CNMA (155 and 240 mg⋅l^-1^) it was recorded as 30.4, 24.8% and 29.95, 30.63%, respectively (data not shown). Necrosis in the presence of ML-CNMA (80, 155, and 240 μg⋅ml^-1^) was observed negligible and recorded as 1.1, 2.2, and 2.8%, respectively. Furthermore, we employed Cyt C release assay to substantiate our apoptotic results. With reference to control cells, the relative percentage of Cyt C release from mitochondria to cytosol in the presence of ML-CNMA (240 mg⋅l^-1^) was increased from 63 to 139%, respectively (**Figure 5B**). The upshot of the increased concentration of Cyt C in the cytoplasm eventually leads to the apoptosis, which corroborates with our TEM and SEM results (**Figure 4**).

### Interaction of CNMA with 14-α Demethylase of *C. albicans*

To further understand a putative mode of action of CNMA, molecular modeling and docking study was performed. Earlier, various molecular mechanism showed the *C. albicans* strains’ resistance against Azoles antifungal agents. The target enzyme of azoles is 14-demethylase (Erg11p), a strategic enzyme in the ergosterol synthesis pathway and block the fungal membrane formation. The overexpression of Erg11p also contributes the fungal resistance (Ramage et al., 2012). CNMA was docked to modeled 14-α demethylase of *C. albicans* (**Figure 5C**), which illustrates the overlapping binding pocket of CNMA with azole drug such as Fluconazole (Supplementary Figure S7). Most of the amino acids involved in the binding interactions were found to be common when tested with CNMA and fluconazole binding with 14-α demethylase (**Figure 5E**). These results were suggestive of the similar mechanistic outcome of CNMA with 14-α demethylase as in the case of azole drugs due to its similar interactions with the targets (**Figures 5D,E**).

### Effect of ML-CNMA on *HWP1* Gene Expression

**FIGURE 6:**
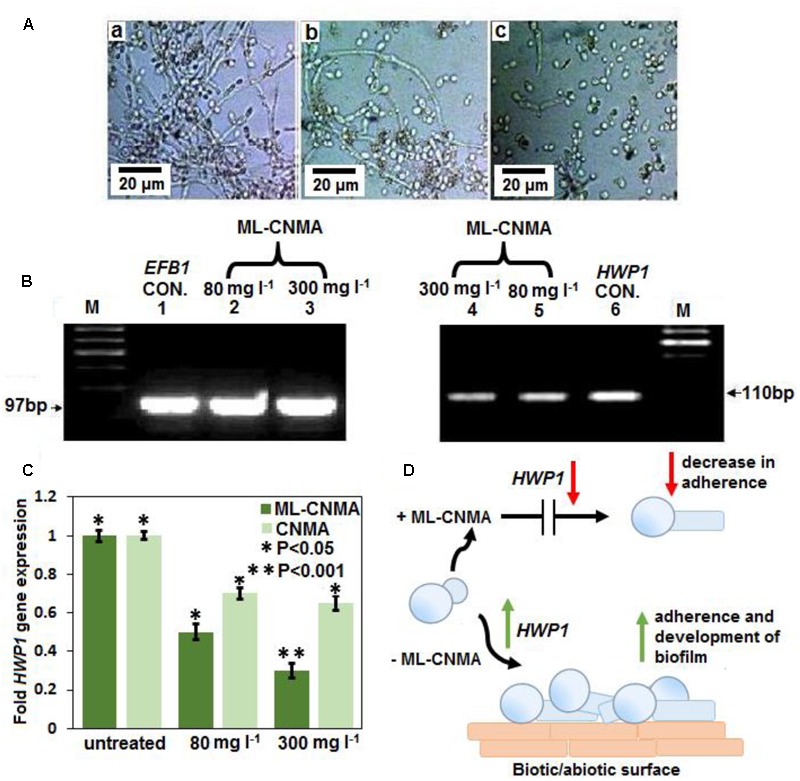
**(A)** Biofilm formation of untreated **(a)** and treated with CNMA **(b,c)** with different concentration (80 and 300 mg⋅l^-1^) for 48 h were observed under light microscope (40×). **(B)** Representative RT-PCR gel images of *EFB1* (lanes 1–3) and *HWP1* (lanes 4–6) transcripts in *C. albicans* cultures grown with increasing concentrations of CNMA at 37°C for 4–6 h. Lane M, marker; lanes 1 and 6, without any treatment (control; CON.); lanes 2 and 5 and 3 and 4, treated with 80 and 300 mg⋅l^-1^, respectively. Panels **(A,B)** shown was from a representative experiment repeated three times with similar results. **(C)** Same RNA samples (as in **B**), were further analyzed with real-time RT-PCR with SYBR green detection system to quantify the expression of *HWP1* gene normalized to a housekeeping gene (*EFB1*). Dissolvent and empty lipid carriers were also tested as controls. Each bar represents the fold change in gene expression (compared with untreated) ± SD of three independent experiments. Error bars indicate the standard error of the mean of three independent experiment performed in triplicate was considered significance, ^∗^*P* < 0.05, ^∗∗^*P* < 0.001, respectively. **(D)** The illustration of decreased in *HWP1* expression corresponding to loss of the adherence and the biofilm development.

In another approach to assessing the effect of ML-CNMA on biofilm formation, *HWP1* gene expression was analyzed. The biofilm formation was initiated by hyphae formation that is imperative to adherence on biotic as well as abiotic surface. The hyphal wall protein (Hwp1) initiates the adherence and biofilm formation. Moreover, inhibition of biofilm formation is directly correlated with HWP1 expression (Nobile et al., 2006). *HWP1* expression was probed when cells were incubated with ML-CNMA (**Figure 6A**), showing the filamentous cells during normal biofilm formation (Hogan et al., 2004). RNA was isolated from cells incubated in the presence (80 and 300 mg⋅l^-1^) and absence of ML-CNMA. Reduction in *HWP1* expression was observed when *Candida* cells were incubated with the increasing concentration of ML-CNMA/CNMA alone (**Figure 6C**). Our data suggests that the gene involved in true hyphal formation was downregulated on treatment with ML-CNMA and leads to decrease in adherence and biofilm initiation (**Figure 6D**). Herein, *EFB1* gene of *C. albicans* was used as a control for uniform expression (housekeeping effect).

## Discussion

To date, several antifungal (Leal et al., 2015) and antibacterial (Gubernator et al., 2007) drugs have been given as encapsulated liposomal formulations to enhance their therapeutic index. In particular, Amp B has been studied exclusively with the aim to reduce its toxicity and side-effects by entrapping into lipid complex and multilamellar (ML) vesicles (Tang et al., 2015). The outermost bilayer of multilamellar phospholipid vesicles fuses with the plasma membrane of the cell, providing increased surface area and invagination of the excess plasma membrane (Batzri and Korn, 1975). In agreement with this, our ML-CNMA results showed more sensitivity than free CNMA are further confirmed by spot test assay (**Figure 1**), which possibly be the result of the sustained release of CNMA in encapsulated form. Earlier it has been reported that fungicidal potential was slightly higher for the multilamellar vesicles than for the small unilamellar vesicles preparation of the tested drugs, such differences were interpreted in terms of lesser release time for the unilamellar aggregates (De Logu et al., 1997). Whereas the lower activity of ML-Amp B than free Amp B against *C. albicans* can be correlated to a study, in which cholesterol does not show any interference with the fungicidal activity. Although, incorporation of Amp B in the ML maintained the antifungal activity against *C. albicans*, but this activity merely not more than free Amp B (Hopfer et al., 1984). This could possibly be the best explanation of lower ML-Amp B activity than free Amp B in our study.

PI has been used to visualize damaged *Candida* cells and Fluorescent Brightener 28 for viewing *Candida* live cells (Harrington and Williams, 2007). The dyes facilitate to observe the interaction between multilamellar lipid vesicles as well as *Candida* cells under a light microscopy (**Figures 2Aa,c**), which were further substantiated by SEM results (**Figures 2Ab,d**). Additionally, these dyes provide an insight on how ML interacts with the fungal cell, employing light microscopy and SEM (**Figures 2Bb,f**). The remarkable reduction in *Candida* biofilm (**Figure 2C**) was observed on treatment with encapsulated ML-forms of CNMA, which was further confirmed by XTT assay (**Figure 3**), illustrating distinct percent inhibition of biofilm formation as compared to untreated controls.

The results of TEM illustrates ML-CNMA induced condensation of chromatin (**Figures 4B,C**), an established marker of yeast apoptosis, indicating the DNA damage caused by ML-CNMA action in *C. albicans*. Moreover, treatment showed a remarkable effect on the cellular surface, with extensive roughening and/or blebbing (**Figure 4D**), further its treatment results in cytological perturbation (**Figures 4F–H**). FUN-1, Con-A, and DAPI fluorescent probes in CLSM micrographs illustrate the decrease of metabolic viability, loss of cell wall integration, and fragmentation or degradation of nuclear DNA (Khan et al., 2012). As ML-CNMA treatment evokes ROS potential in *Candida* cell (**Figure 4J**), which suggests ROS driven cellular injury.

**FIGURE 7:**
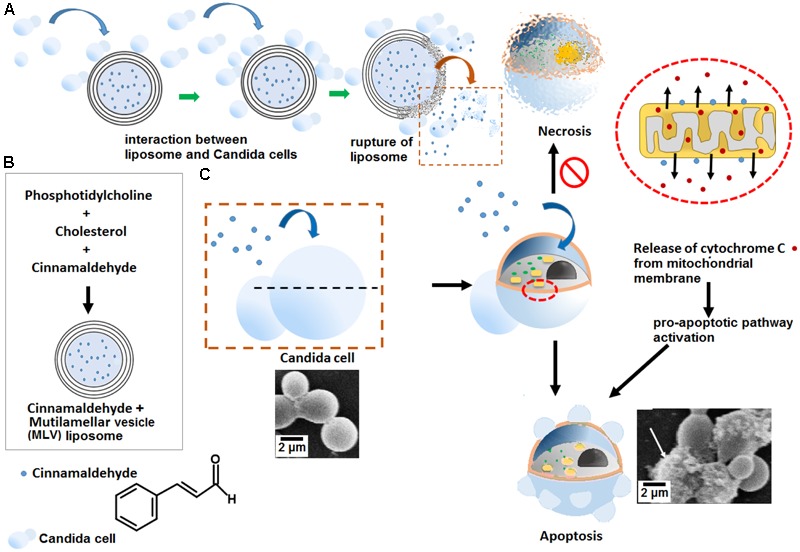
Illustrative schematic mechanism of ML-CNMA-induced apoptosis in *C. albicans*. **(A)** ML liposome interacts with *C. albicans* and leads to rupture of ML-CNMA and finally releases of CNMA. **(B)** This liposome has trapped CNMA and **(C)** after the rupture of liposomes, CNMA induced the elevation of mitochondrial potential, causing ROS accumulation in mitochondria, and resulting in the release of Cyt C to the cytosol. Both ROS and Cyt C activated the pro-apoptotic pathway, which may trigger apoptosis in *C. albicans*.

Furthermore, ML-CNMA showed better antibiofilm activity (**Figures 2C, 3**) and negligible necrosis as shown in our FACS analysis (**Figure 5A**) suggesting its use with lesser unspecific toxicity. As here, we observed only early apoptosis and a negligible amount of late apoptosis in the presence of ML-CNMA, whereas free CNMA showed approximately same early and late apoptosis (data not shown), which further corroborates with our spot test results (**Figure 1**). Moreover, to elucidate the possible role of ML-CNMA, Cyt C release assay was further performed (**Figure 5B**). The results illustrate ML-CNMA induced mitochondrial hyperpolarization in *C. albicans* (Wu et al., 2009) and possible induction of apoptotic pathway (Desagher and Martinou, 2000). This increase in Cyt C concentration in cytosol evokes ROS generation, which is also a proapoptotic event (De Logu et al., 1997). Hence, this implicates that the ML-CNMA extract may induce apoptosis through the metacaspase-dependent apoptotic pathway, which is activated by Cyt C release and ROS production, as depicted in the suggestive scheme (**Figure 7**).

When probed for gene expression, results illustrates repression of the gene implicated in biofilm formation and true hyphal formation on ML-CNMA treatment and thereby inhibits the process of biofilm formation, which further corroborates with our SEM results in **Figure 2**. Earlier studies were also in accordance with our findings by suggesting a correlation between *HWP1* expression and biofilm formation (Nobile et al., 2006). Molecular modeling and docking studies revealed molecular interaction involved of CNMA with 14-α demethylase of *Candida* (**Figure 7**). The inhibition of fungal cell wall synthesizing enzymes by *trans*-CNMA has also been studied earlier (Bang et al., 2000). Which is a cell wall active antifungal agent that acts as a potentiator by reducing the cell wall synthesis and facilitating the leakage of fungal cytoplasm (Yen and Chang, 2008). Furthermore, our results (**Figures 5C–E**) suggest the binding of CNMA at the active site of the enzyme and amino acid residues involved in the interactions of CNMA are overlapping to azole binding drugs (Baginski and Czub, 2009). Hence, a similar interaction of CNMA as of azole drugs is suggested upon its treatment (Paranagama et al., 2010).

Evidence regarding mitochondrial involvement in the induction of yeast apoptosis in response to different stimuli has been well established (Ludovico et al., 2002). Our FACS analysis (**Figure 6A**) release of Cyt C (**Figure 6B**) data and TEM data (**Figure 4**) showed the apoptotic effect in the presence of ML-CNMA and is strongly supported by the crucial role of mitochondria in cell death induced by hyperosmotic stress (Silva et al., 2005). This study concludes the potential use of ML-CNMA as an antifungal and antibiofilm agent. The findings of this study demonstrate that ML-CNMA retards the proliferation of *C. albicans* and promotes apoptosis. This is the first study, using ML-CNMA extract to induce apoptosis in *C. albicans* and hence suggesting its potential use against *Candida* infections in immune compromised patients. Moreover, this study also recommends the multilamellar liposomal preparation of antifungal compounds to increase the efficacy of antifungal agents.

## Author Contributions

AK, SK, and SNK designed the experiments. SK, SNK, JI, and RK performed the experiments. AK, SNK, and SK wrote the paper. AK and SNK reviewed the manuscript. All authors reviewed the paper.

## Conflict of Interest Statement

The authors declare that the research was conducted in the absence of any commercial or financial relationships that could be construed as a potential conflict of interest.
