# Supplementary material for: Enhanced Killing and Antibiofilm Activity of Encapsulated Cinnamaldehyde against Candida albicans
Source: Front Microbiol. 2017 Aug 29;8:1641. doi: 10.3389/fmicb.2017.01641 (PMC5581813; doi:10.3389/fmicb.2017.01641)
Supplement: Supplementary file 1 [file Data_Sheet_1.PDF]

Supplementary information

Enhanced killing and antibiofilm activity of encapsulated cinnamaldehyde (CNMA) against *Candida albicans*

Shahper N. Khan[ǂ], Shakir khan[ǂ], Jawed Iqbal, Rosina Khan and Asad U. Khan*

Affiliation

Interdisciplinary Biotechnology Unit, Aligarh Muslim University Aligarh, 202002 India,

Corresponding author: *Asad U. Khan, Professor, Interdisciplinary Biotechnology Unit, Aligarh Muslim University, Aligarh-202002, India.

Email: asad.k@rediffmail.com

Telephone: 00919837021912

Fax: 0091-571-2721776

Table of contents	Page no.
**Supplementary figures**	2-8
**Supplementary Fig. S1:** The concentration based absorbance spectra of amphotericin B…………………………………………………………………..	2
**Supplementary Fig. S2:** The concentration based CFU. of CNMA alone calibration graph for calculations of multilamellar liposome entrapped CNMA…….	3
**Supplementary Fig. S3:** The size distribution of ML-cinnamaldehyde…………..	4
**Supplementary Fig. S4:** The light microscopic image of the synthesized (300 mg l^{-1} CNMA entrapped) ML-CNMA……………………………………………………	4
**Supplementary Fig. S5:** The size distributions or histograms (based on the SEM images) of ML-CNMA………………………………………………………………	5
**Supplementary Fig. S6:** Reduction in biofilm formation was assessed by XTT and Crystal violet assay….. by ML-Amp B and Amp B………………………………	7
**Supplementary Fig. S7:** The interaction of Fluconazole with active site of 14-α demethylase………………………………………………………………………..	8
**Supplementary tables**	
**Supplementary Table S1:** The relations between the effective percentage viability (E) at the effective doses of CNMA and ML-CNMA on various *C. albicans*………..	5
**Supplementary Table S2:** The anti-biofilm activities of the CNMA alone and ML-CNMA on the 24 and 48 hours grown various *C. albicans* biofilms………………..	6

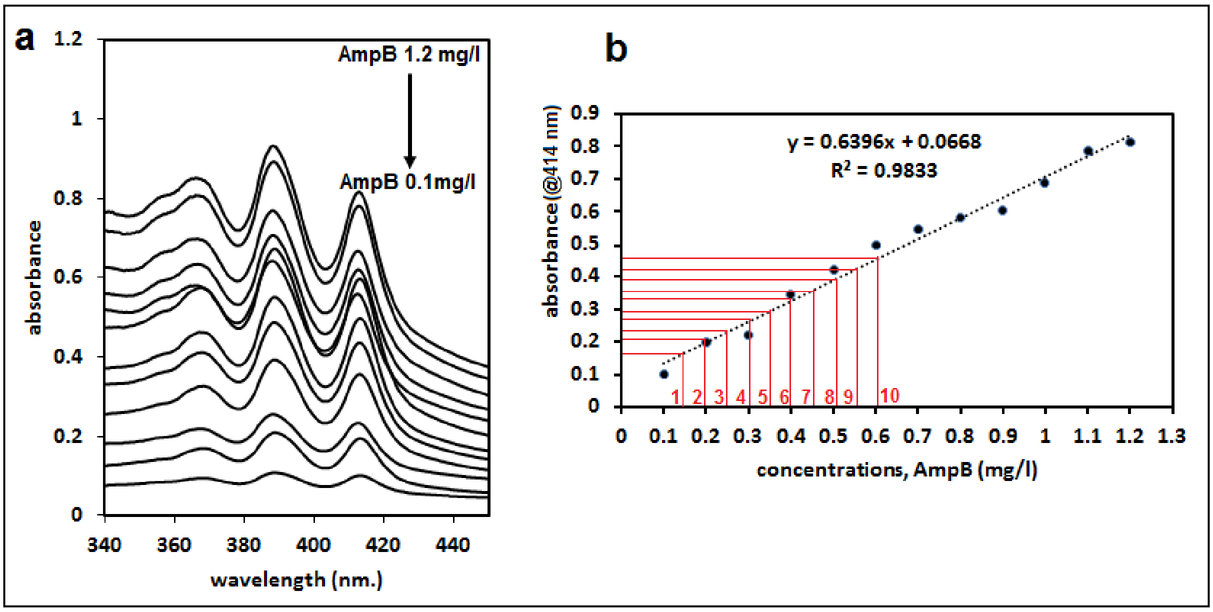

Fig. S1: (a) The concentration based absorbance spectra of Amp B and calibration graph **(b)** for calculations of multilamellar liposome entrapped amphotericin B (ML-AmpB; 1=0.15±0.02, 2=0.20±0.03, 3=0.25±0.06, 4=0.30±0.05, 5=0.35±0.03, 6=0.40±0.06, 7=0.45±0.05, 8=0.52±0.01, 9=0.56±0.04 and 10=0.61±0.03 mg/l)

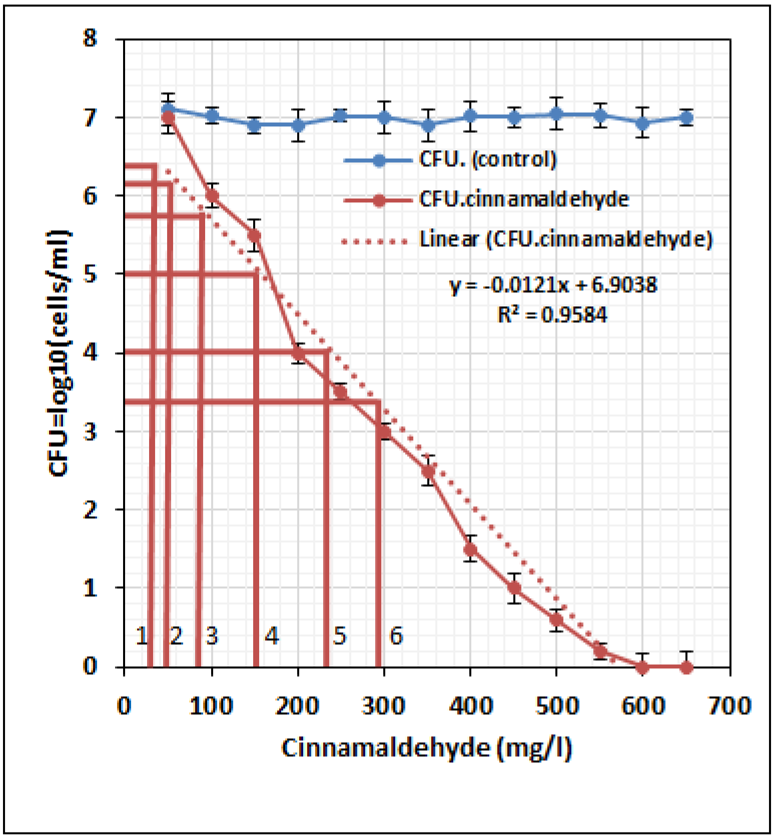

Fig. S2: The concentration based CFU. of CNMA alone calibration graph for calculations of multilamellar liposome entrapped CNMA (ML-CNMA; 1=30±2.6, 2=56±2.0, 3=80±4.1, 4=155±4.2, 5=240±1.1, and 6=300±5.7mg/l).

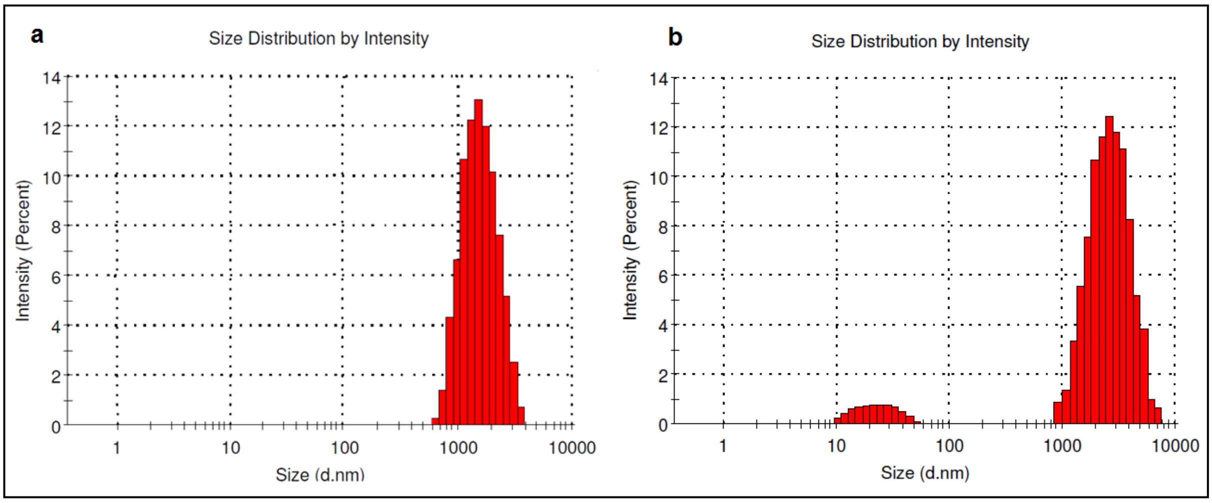

Fig. S3: The size distribution of 0.45mg l^{-1} Amp B entrapped ML-Amp B. (a) and 300 mg l^{-1} CNMA entrapped ML-CNMA (b). **Result**: The size of the synthesized ML-Amp B is 1.9±2µm and ML-CNMA has 2.7±3µm size.

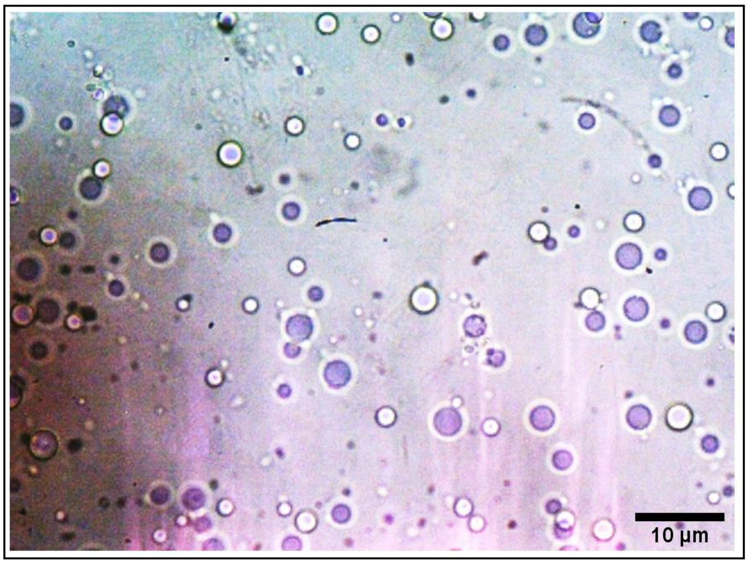

Fig. S4: The light microscopic image of the synthesized (300 mg l^{-1} CNMA entrapped) ML-CNMA liposomes (mean size= ~ 2.7µm).

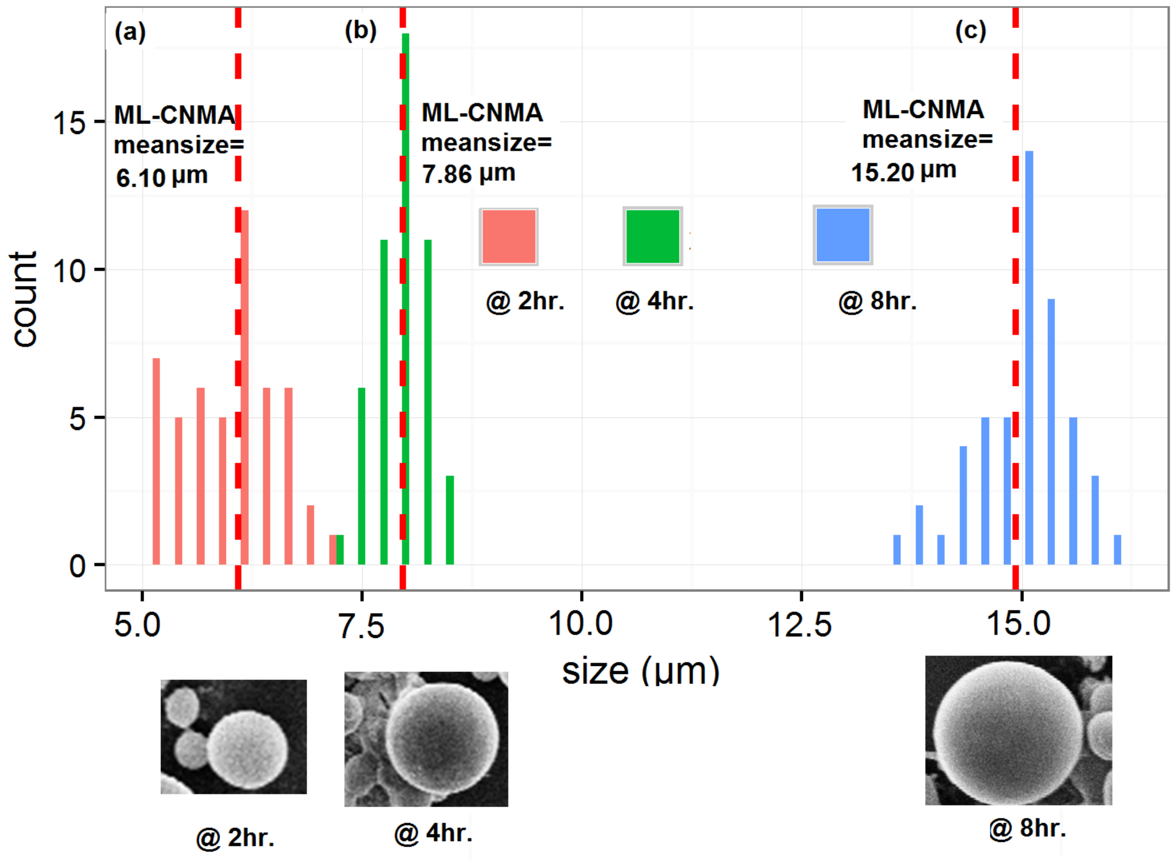

Fig. S5: The size distributions or histograms (based on the SEM images) of ML-CNMA (300 mgl^{-1})calculated by ImageJ software (ImageJ 1.49 v, http://imagej.nih.gov/ij/download.html). The SEM images were taken at 2, 4 and 8 hours incubations (after the initial treatment with ML-CNMA on *C. albicans.*

Table S1: The relations between the effective percentage viability (*E*) at the effective doses of CNMA and ML-CNMA on various *C. albicans*.

strains	*E* value	Effective dose for CNMA only (µg/ml ±S.E.)	Effective dose for ML-CNMA (µg/ml ±S.E.)
CA03	*E50*	401.3±05	62.1±4.0
	E99	1153.0±16	160.0±3.3
CA05	*E50*	325.0±30	56.2±.6.5
	E99	1193±15	231±1.2
CA011	*E50*	673±12.0	170±4.5
	E99	2480±14.6	310±2.0
CA16	*E50*	511.7±11	146.6±0.6
	E99	1205±12	208.7±5.0
CA*	*E50*	332.9±3.7	56.3±1.4
	E99	571.8±24.7	162.4±13.4

* *Candida albicans* ATCC24433

Table S2: The anti-biofilm activities of the CNMA alone and ML-CNMA on the 24 and 48 hours grown various *C. albicans* biofilms.

drugs	treatment	biofilm reduction									
		% biofilm^Φ^ reduction of					% Viable biofilm*reduction of				
		ATCC$	CA11	CA05	CA03	CA16	ATCC$	CA11	CA05	CA03	CA16
CNMA@	on 24 h grown biofilm	71.8	44.1	39.9	45.0	21.7	25.8	30.3	31.0	27.2	34.3
	on 48 h grown biofilm	76.0	66.3	32.4	35.7	36.0	31.3	24.2	27.0	22.0	20.4
ML-CNMA@	on 24 h grown biofilm	81.3	93.6	65.2	80.6	75.3	55.0	59.7	60.6	54.1	53.7
	on 48 h grown biofilm	83.2	89.8	66.6	84.0	74.0	68.9	58.0	57.0	50.7	50.4

@The sub MIC concentrations; i.e. 500 mg/l for CNMA alone and 300 mg/l for ML-CNMA.

Φ The mean values of percentage biofilm reduction (values determined by Crystal violet biofilm reduction assay, n=3).

* The mean values of percentage viable biofilm reduction (values determined by XTT biofilm reduction assay, n=3).

$ *Candida albicans* ATCC24433.

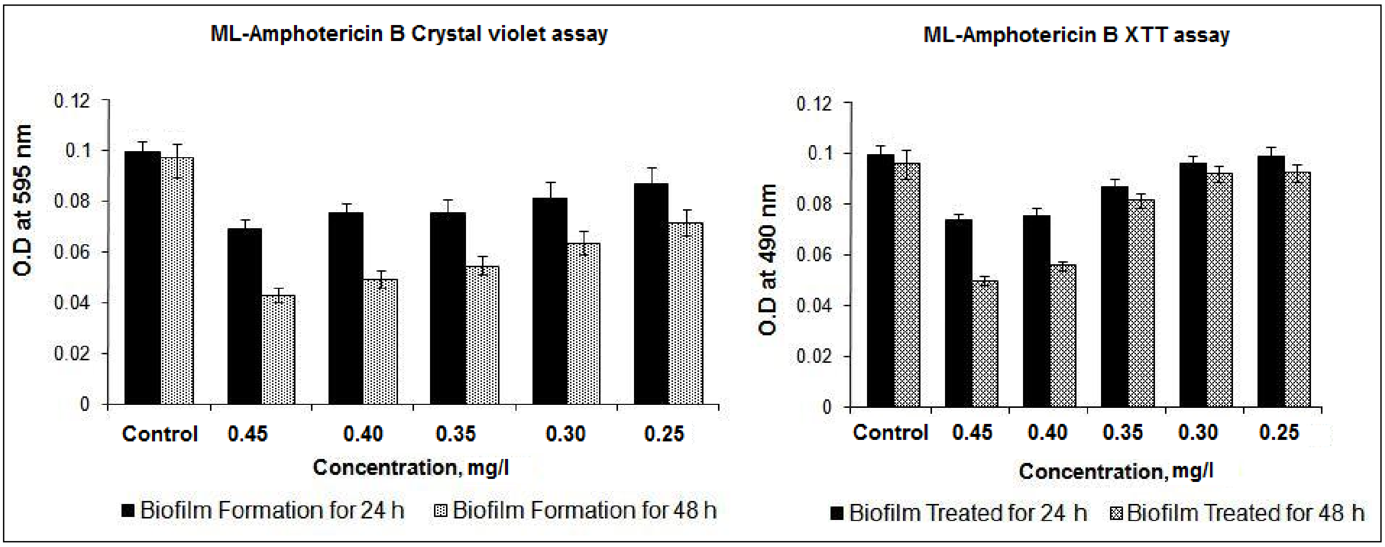

Fig. S6: Reduction in biofilm formation was assessed by XTT and Crystal violet assay. In XTT and CV assay, candida biofilm (ATCC 24433) was treated with decreasing concentration of Amp B (0.45, 0.40, 0.35, 0.30 and 0.25 mg.l^{-1}) @/in ML-Amp B, the results were normalized with untreated sample. Control (untreated) and treated biofilm were grown for 24 & 48 hrs. The final values were mean optical density (OD) at 490 and 595 nm, respectively. Error bars indicate the standard error of the mean of three independent experiments performed in triplicates.

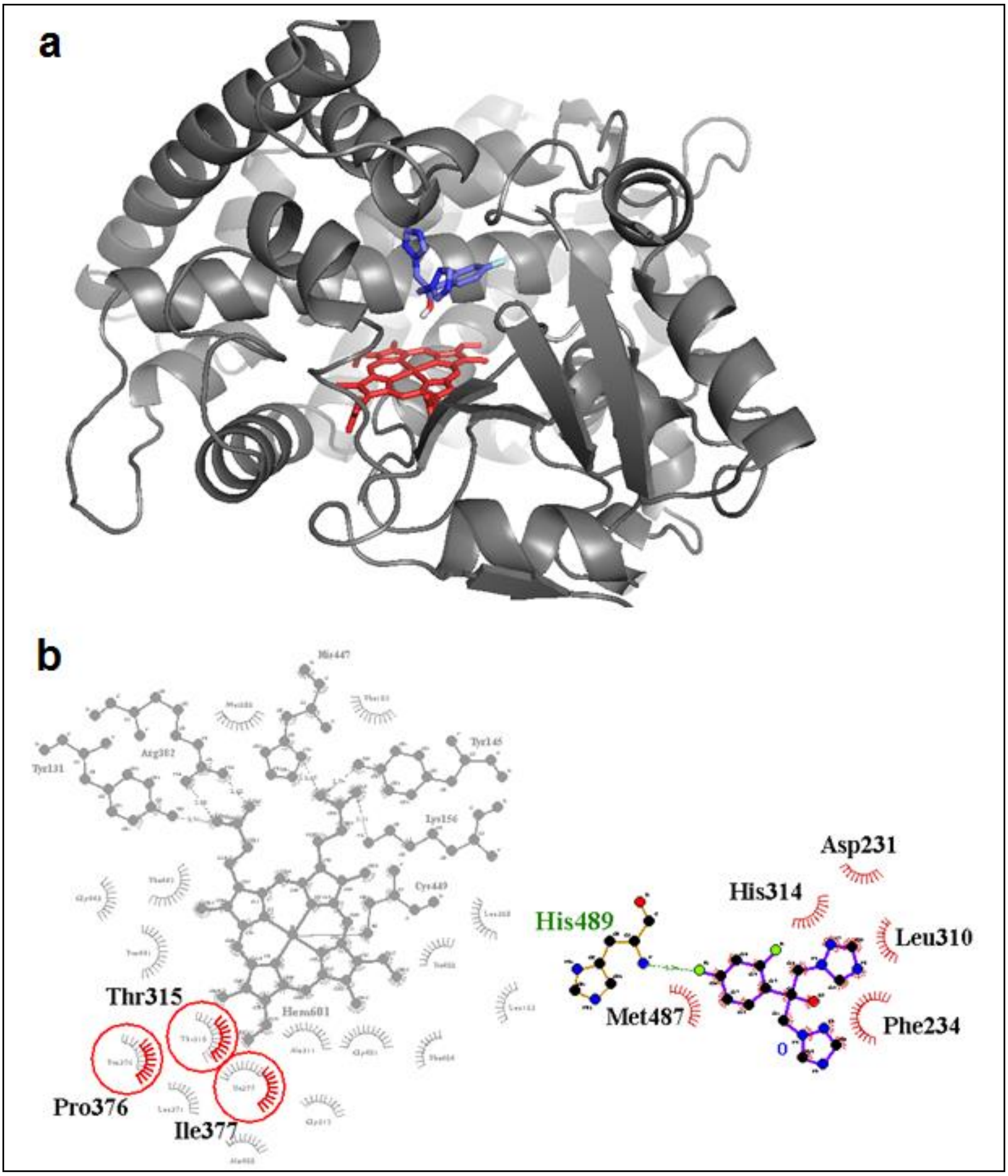

Fig. S7: **(a)** The interaction of Fluconazole (PubChem CID: 3365) with active site of 14-α demethylase (3D model in PyMol GUI). The amino acids involved as hydrophobic interactions (Thr 315, Pro376 and Ile377) with Hem601, and these amino acids also involved in the same interactions with Fluconazole shown in LigPlot (executed in LIGPLOT+, Version v.1.4.5) (**b**).
